# A231 THE FLAVANOID CYANIDIN REDUCES INTESTINAL INFLAMMATORY DAMAGE BY MAINTAINING INTESTINAL PERMEABILITY

**DOI:** 10.1093/jcag/gwac036.231

**Published:** 2023-03-07

**Authors:** R D C Santos, C Baggio, W K MacNaughton

**Affiliations:** 1 Laboratory of Pharmacology and Molecular Biology, São Francisco University, Bragança Paulista, Brazil; 2 Department of Physiology and Pharmacology and Snyder Institute for Chronic Diseases, University of Calgary, Calgary, Canada

## Abstract

**Background:**

Since the discovery and the use of proton pump inhibitors (PPI), the incidence of gastric ulcers has reduced, however, there has been an increase in intestinal lesions since PPIs do not guarantee protection to the intestine. To protect the deeper layers of the intestinal wall, the intestinal barrier tightly regulates the passage of pro-inflammatory molecules, microorganisms, toxins, and antigens. The epithelium is damaged during intestinal inflammation due to the action of inflammatory cytokines such as TNF resulting in ulceration. Cyanidin is a flavonoid of the anthocyanin class, found in several red fruits, with anti-inflammatory and antioxidant activity.

**Purpose:**

We hypothesized that the administration of cyanidin can decrease the deleterious effects caused by polypharmacy (PPI + NSAID) in the small intestine by reducing intestinal permeability.

**Method:**

*In vivo*: Male mice (8 weeks old) were treated to induce intestinal ulcers caused by polypharmacy (combination of PPI - lansoprazole 20 mg/kg daily, non-steroidal anti-inflammatory drug (NSAID) - acetylsalicylic acid 10 mg/kg daily and COX-2 selective NSAID, celecoxib 20 mg/kg daily). On the ninth day, oral treatment with cyanidin (5 mg/kg) or vehicle was started until the fourteenth day, then the animals were killed for parameter analysis lesion. ELISA was performed to quantify interleukins (IL-10, IL-6, IL-1β) and cytokine (TNF), and we assessed the antioxidant profile (SOD, CAT, and GSH) and measured gene expression of TNF, IL-10, IL-6 TRL-4, HMOX-1, MMP 2 and 9, COX-1, MUC-3, ZO-1, CL-1. *In vitro*: Monolayers of colonic epithelial cell lines (Caco-2 at 21 days of confluence) were mounted in Ussing chambers to assess barrier function and to determine transepithelial resistance (TER). To analyze the permeability response to injury, we utilized TNF and IFN (25 ng/mL) with cyanidin (10 or 100 uM) for 48 h in transwell plates, with measurement of total intestinal permeability using 4 kD FITC-dextran.

**Result(s):**

Analysis of mouse intestine indicated that cyanidin (5 mg/kg) significantly reduced expression of IL-6 and TNF, TLR4, and HMOX-1 (p<0.05), and increased gene expression of MUC-3, CL-1, occludin, COX-1, and IL-10 (p<0.05) without altering antioxidant parameters. Cyanidin (100 mM) maintained barrier function as shown by transepithelial electrical resistance (TER), and also significantly reversed the detrimental effects of the inflammatory cytokine on FITC-dextran flux in Caco-2 cells (p<0.05). This may be related to the increased expression of occludin and ZO-1 in the intestinal epithelium of mice.

**Conclusion(s):**

These *in vivo* and *in vitro* results suggest that cyanidin decreases the polypharmacy-induced intestinal inflammatory response while maintaining the integrity of the intestinal epithelium. Other trials are underway to elucidate these mechanisms.

**Please acknowledge all funding agencies by checking the applicable boxes below:**

Other

**Please indicate your source of funding;:**

NSERC; FAPESP

**Disclosure of Interest:**

None Declared

